# Insulin levels at 18–20 gestational weeks in pregnant women with obesity are associated with newborn abdominal fat deposition and DNA methylation in cord blood

**DOI:** 10.1186/s13148-025-01923-y

**Published:** 2025-07-11

**Authors:** Alice Maguolo, Josefine Jönsson, Alexander Perfilyev, Allan Vaag, Emma Malchau Carlsen, Kirsten Nørgaard, Paul W. Franks, Kristina M. Renault, Charlotte Ling

**Affiliations:** 1 Epigenetics and Diabetes Unit, Department of Clinical Sciences in Malmö, Lund University Diabetes Centre, Scania University Hospital, Malmö, Sweden; 2 Department of Clinical Sciences in Malmö, Lund University Diabetes Centre, Scania University Hospital, Malmö, Sweden; 3 Copenhagen University Hospital–Steno Diabetes Center Copenhagen, Borgmester Ib Juuls Vej 83, 2730 Herlev, Denmark; 4 Department of Nutrition, Exercise and Sports, Faculty of Science, University of Copenhagen, Frederiksberg, Denmark; 5 Department of Neonatology, Copenhagen University Hospital Rigshospitalet, Copenhagen, Denmark; 6 Department of Clinical Medicine, Faculty of Health and Medical Sciences, University of Copenhagen, Blegdamsvej 3B, 2200 Copenhagen N, Denmark; 7 Department of Nutrition, Harvard T.H. Chan School of Public Health, Boston, MA USA; 8 Department of Obstetrics, Juliane Marie Centre, Rigshospitalet, University of Copenhagen, Copenhagen, Denmark

**Keywords:** Epigenetics, DNA methylation, Diabetes, Fetal programming, Early life, Maternal insulin, Body composition, Abdominal adiposity

## Abstract

We assessed if fasting plasma insulin levels in pregnant women with obesity are associated with newborns’ abdominal fat deposition (dual-energy X-ray absorptiometry) and with cord blood DNA methylation (450k array) in 232 mother–child pairs from the Treatment of Obese Pregnant women (TOP) study. Fasting maternal insulin at 18-20gw was associated with abdominal/total fat mass ratio in newborns independent of multiple potential confounders (*β* = 0.23[95%CI: 0.01; 0.45], *P* = 0.041) and with cord blood DNA methylation at CpG sites annotated to *C11orf54* and *RARB* (FDR < 10%), both genes potentially involved in metabolic programming. In conclusion, maternal insulin levels in pregnancy were associated with adiposity traits and epigenetics in the offspring.

**Supplementary Information:**

The online version contains supplementary material available at 10.1186/s13148-025-01923-y.

## Introduction

Maternal metabolic health during pregnancy may impact offspring metabolic programming through epigenetic mechanisms [1]. Early abdominal fat deposition is likely to track throughout life, being an independent risk factor for type 2 diabetes (T2D) and adverse cardiometabolic health [2]. Although maternal obesity, gestational weight gain (GWG), and gestational diabetes mellitus (GDM) have been associated with fetal growth and neonatal total fat deposition [3, 4], little is known so far regarding the early determinants of abdominal fat distribution from birth. This is compounded by the dearth of reliable measures of neonatal abdominal adiposity [5].

The Treatment of Obese Pregnant women (TOP) study offers a rare opportunity to investigate the link between maternal pregnancy risk factors and early abdominal adiposity in newborns, assessed through dual-energy X-ray absorptiometry (DXA) scan [6]. We previously reported that an increase in abdominal fat mass of newborns was linked to pre-pregnancy BMI (prePregBMI), GWG, and 2-h glucose at 27–30 gestational weeks (gw) [6]. Glucose readily crosses the placenta and induces fetal hyperinsulinemia, which has an anabolic action enhancing fetal growth and fat accretion [7]. The prevalence of overweight and obesity in women entering pregnancy has increased worldwide [ ^8^,^9^]. Pregnancy induces insulin resistance, which is further increased in women with obesity, resulting in hyperinsulinemia [10, 11]. The known correlation between prePregBMI and newborn body composition may be mediated through early maternal metabolic imbalances to which insulin resistance is a main contributor [7, 11, 12]. The potential independent role of early maternal insulin levels on placenta metabolism and fetal adiposity was recently summarized [7, 12–14]. Even if insulin does not cross the placenta, the placenta has abundant insulin receptors and maternal insulin can activate signaling pathways affecting placental metabolism, even in euglycemic conditions, and thereby influencing placental and fetal development [7, 11]. Gestational insulin resistance was reported as a predictor of neonatal and infant adiposity [10, 13–15], independent of prePregBMI or GWG [10, 14, 16]. Previous studies have linked maternal insulin or glucose to placental or cord blood DNA methylation (DNAm) [17–19]. However, to the best of our knowledge, the impact of maternal plasma insulin levels at 18-20gw on newborn abdominal adiposity and cord blood DNAm has not been investigated.

Thus, our aim was to assess if fasting plasma insulin levels at 18-20gw in pregnant women with obesity from the TOP study are associated with abdominal fat deposition (i.e., abdominal/total fat mass ratio (FMr)) in newborns, independent of other maternal risk factors, including prePregBMI and GWG. Moreover, as DNAm is a proposed mechanism by which maternal risk factors may predispose offspring to cardiometabolic disease throughout life [1], we aimed to investigate associations between maternal insulin levels and cord blood DNAm.

## Methods

**Fig. 1:**
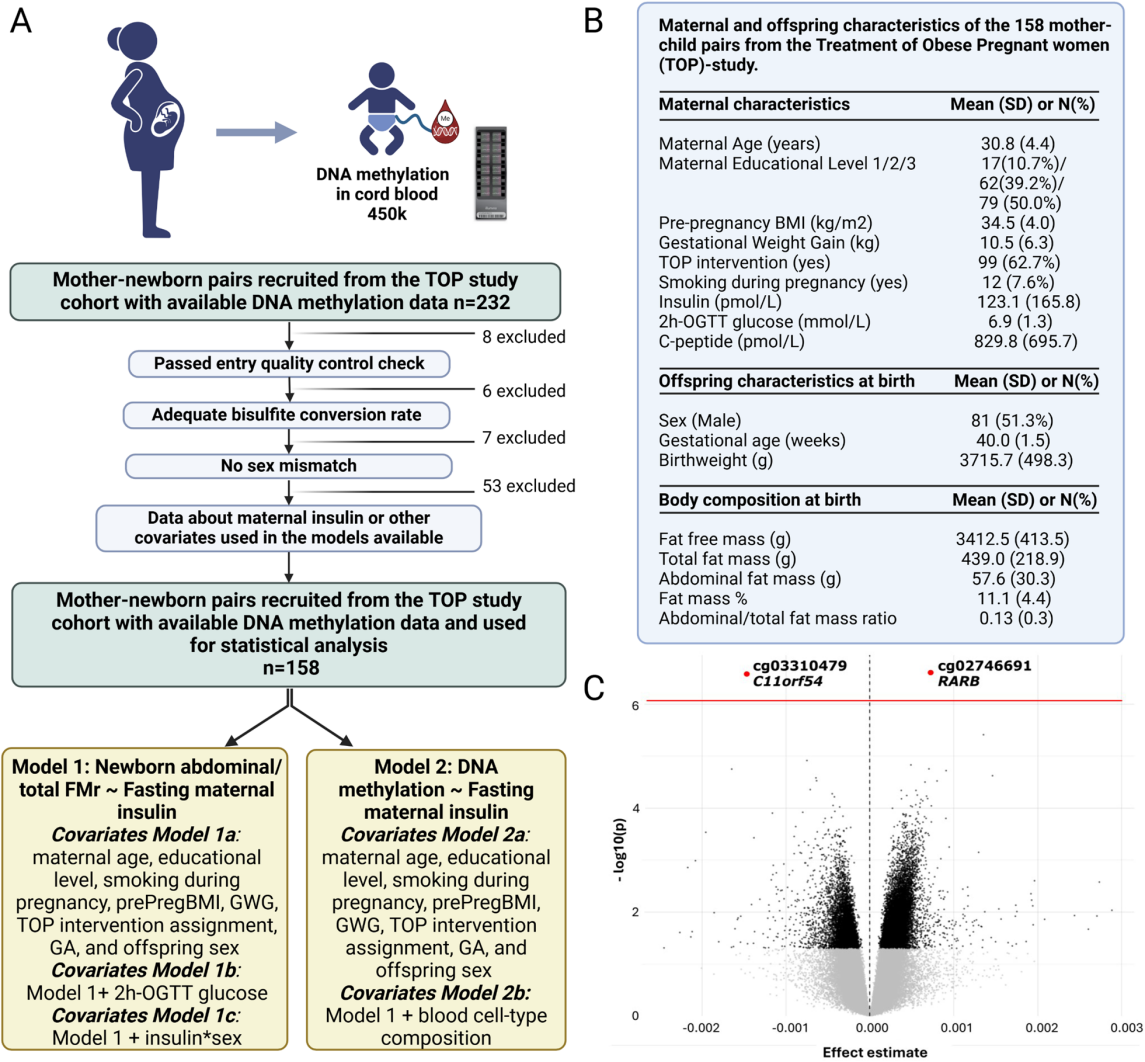
**A** Study design and flow-chart for the samples selection of the mother–child pairs included in this study from the Treatment of Obese Pregnant women (TOP) study (n = 232). **B** Maternal and offspring characteristics of the mother–child pairs included in the statistical analysis from the TOP study (n = 158). Created with Biorender. **C** Volcano plot showing the associations between DNA methylation and maternal insulin, adjusted for maternal age, educational level, smoking during pregnancy, pre-pregnancy BMI, gestational weight gain, TOP intervention assignment, gestational age, offspring sex, and cell-type composition. A total of 23,204 CpG sites with *P* < 0.05 are shown in black, while the 2 CpG sites with FDR < 10% are highlighted in red. Created with Biorender

Two hundred thirty-two mother–child pairs of European ancestry with available cord blood DNAm data from the TOP study were included (Fig. 1A). The TOP study is a randomized controlled trial (RCT) of 425 pregnant women with obesity assigned to lifestyle interventions including diet and physical activity [6, 20], approved by the Ethics Committee for the Capital Region of Denmark (H–D-2008–119), and registered at ClinicalTrials.gov (NCT01345149). The present study is based on previously collected biological samples and anthropometrics data [20]. Information on recruitment, data collection, and trial conduct is described elsewhere [6, 20]. The maternal and offspring measurements described in Fig. 1B were considered for this study including the abdominal/total FMr, calculated as abdominal fat mass (g) / total fat mass (g), assessed by DXA scan in newborns within 48 h from birth. Maternal 2-h glucose was assessed at 18-20gw through an oral glucose tolerance test (OGTT), and fasting samples of insulin and c-peptide were collected. Detailed information is available in Supplementary File. Genome-wide DNAm analysis was undertaken in cord blood using Infinium HumanMethylation450 BeadChips (Illumina, San Diego, CA) as previously described [21]. Methylation data were acquired from 460,729 probes. We filtered out 1594 probes with mean detection *P*-value ≥ 0.01, 65 rs-probes, 3,091 ch-probes targeting non-CpG sites, 416 Y-chromosome probes, 14,466 cross-reactive probes, and 5,216 polymorphic probes with a minor allele frequency > 0.1. The bioinformatics pipeline is described in Supplementary File, including the reference-based method employed to correct for cell-type composition heterogeneity and batch effects adjustment. Statistical analyses were conducted in data from 158 mother–child pairs, after excluding samples that failed DNAm quality control, had inadequate bisulfite-conversion rate, showed sex mismatch, and/or lacked data regarding maternal insulin or covariates used in the statistical models (Fig. 1A). For statistical analyses, β-values were converted into M-values to eliminate heteroscedasticity (M = log2(β/[1 − β]). Spearman and Pearson’s correlation were used to assess bivariate associations (Supplementary File). Multivariable linear regression was used to assess associations between maternal insulin levels and newborn abdominal/total FMr, adjusting for maternal age, educational level, smoking during pregnancy, prePregBMI, GWG, TOP intervention assignment, gestational age (GA), and offspring sex (Model 1a) + 2 h-OGTT glucose (Model 1b) (Fig. 1A). Since sex differences in the associations between maternal metabolism and neonatal adiposity have been reported [13, 14], potential differences across sex were explored using an interaction term (insulin*sex) (Model 1c, Fig. 1A) (Supplementary File). Additionally, associations between maternal insulin and cord blood DNAm were assessed, adjusting for the same covariates in Model 2a + cell-type composition (Model 2b) (Fig. 1A). Associations between maternal insulin and cord blood DNAm were corrected for multiple testing using Benjamin–Hochberg’s method, and associations with false discovery rate (FDR) < 10% are considered. Statistical analyses were performed using R software (v.4.2.1) and IBM SPSS Statistics (v29). Gtex Portal (https://gtexportal.org/home), GWAS Catalog (https://www.ebi.ac.uk/gwas), EWAS Catalog (https://www.ewascatalog.org/), EWAS Atlas (https://ngdc.cncb.ac.cn/ewas/atlas/index), PubMed (https://www.ncbi.nlm.nih.gov), and JASPAR database [22] through UCSC Genome Browser (https://genome.ucsc.edu) were searched for assessing the biological relevance of DNAm sites and annotated genes associated with maternal insulin.

## Results

The clinical and biochemical characteristics of mothers and offspring are described in Fig. 1B. In bivariate correlation analyses, abdominal/total FMr correlated both with maternal insulin and c-peptide levels at 18-20gw [ρ = 0.22 (*P* = 0.023) and ρ = 0.20 (*P* = 0.032), respectively]. As insulin and c-peptide levels correlated strongly (ρ = 0.89, *P* < 0.001), indicating robustness of both markers, subsequent analysis was pursued using insulin. Maternal insulin levels were significantly associated with the newborn abdominal/total FMr, independent of maternal age, educational level, smoking during pregnancy, prePregBMI, GWG, TOP intervention assignment, offspring sex, and GA (β = 0.24[95%CI:0.03;0.45], *P* = 0.028) (Model 1a, Table 1). This association remained after adjusting the same model for the 2 h-OGTT glucose levels (β = 0.23[95%0.01;0.45], *P* = 0.041) (Model 1b, Table 1). This means that each 1-SD increase in maternal insulin corresponds to a 0.23–0.24 SD increase in abdominal/total FMr, after controlling for the other factors. The interaction term insulin*sex in Model 1c was not statistically significant, indicating no evidence that the association between insulin and abdominal/total FMr differs by sex (Table 1).

**Table 1:** Linear regression analysis to assess the association between maternal insulin at 18–20 gw and newborn abdominal/total FMr, independent of maternal age, educational level, smoking during pregnancy, pre-pregnancy BMI, gestational weight gain, TOP intervention assignment, gestational age, and offspring sex (Model 1a) + 2 h-OGTT glucose (Model 1b). In Model 1c interaction between sex and maternal insulin has been tested

	Standardized estimate	Estimate	*p*-value	95% Confidence interval for estimate	
				Lower bound	Upper bound
*Model 1a*					
Insulin (pmol/l)	0.235	4.49E-05	0.028	4.87E-06	8.49E-05
Maternal age (years)	− 0.001	− 3.45E-06	0.996	− 0.001	0.001
Smoking (yes)	0.041	0.004	0.681	− 0.014	0.021
Education^§^	0.071	0.001	0.511	− 0.002	0.005
PrePregBMI (kg/m^2^)	0.062	4.03E-04	0.568	− 0.001	0.002
GWG (kg)	0.052	2.42E-04	0.611	− 0.001	0.001
Gestational age (weeks)	0.068	0.001	0.496	− 0.003	0.006
Sex (M)	0.132	0.007	0.181	− 0.003	0.018
TOPs intervention (yes)	− 0.122	− 0.007	0.219	− 0.019	0.004
*Dependent variable: abdominal-total fat mass ratio*					
Model 1b*					
Insulin (pmol/l)	0.229	4.28E-05	0.041	1.75E-06	8.39E-05
Maternal age (years)	− 0.009	− 5.71E-05	0.936	− 0.001	0.001
Smoking (yes)	0.009	0.001	0.937	− 0.018	0.019
Education^§^	0.069	0.001	0.539	− 0.003	0.005
PrePregBMI (kg/m^2^)	0.022	1.48E-04	0.843	− 0.001	0.002
GWG (kg)	− 0.001	− 2.83E-06	0.996	− 0.001	0.001
Gestational age (weeks)	0.010	0.000	0.926	− 0.005	0.005
Sex (M)	0.130	0.007	0.207	− 0.004	0.019
TOPs intervention (yes)	− 0.127	− 0.007	0.220	− 0.019	0.005
2 h-OGTT glucose (mmol/L)	− 0.138	− 0.003	0.239	− 0.007	0.002
*Dependent variable: abdominal-total fat mass ratio*					
*Model 1c*^*#*^					
Insulin (pmol/l)	0.245	4.67E-05	0.044	1.33E-06	9.20E-05
Maternal age (years)	− 0.001	− 4.21E-06	0.995	− 0.001	0.001
Smoking (yes)	0.039	0.003	0.700	− 0.014	0.021
Education^§^	0.070	0.001	0.525	− 0.003	0.005
PrePregBMI (kg/m^2^)	0.060	3.94E-04	0.580	− 0.001	0.002
GWG (kg)	0.056	2.60E-04	0.596	− 0.001	0.001
Gestational age (weeks)	0.067	0.001	0.509	− 0.003	0.006
Sex (M)	0.116	0.007	0.386	− 0.008	0.021
TOPs intervention (yes)	− 0.121	− 0.007	0.225	− 0.019	0.004
Insulin*sex	− 0.024	− 7.89E-06	0.866	− 1.01E-04	8.49E-05
*Dependent Variable: abdominal-total fat mass ratio*					

^*^Model 1b: same covariates Model 1a + 2 h-OGTT glucose;

^#^ Model 1c: same covariates Model 1a + interaction factor (insulin*sex)

^*§*^Education: Maternal educational level was categorized into three categories: 1) Grammar school 10 years; 2) Secondary school 12 years, Vocational training school, and Further education 1–2 years; 3) Tertiary education 3–4 years (Bachelor level) and Advanced education (post-graduate)

Variance inflation factor (VIF) values of the Models 1a and 1b = 1.053–1.359. VIF provides a measure of multicollinearity among the independent variables in a multiple regression model. These values indicate mild or negligible multicollinearity among predictor variables

*CI* confidence interval, *GWG* gestational weight gain, *OGTT* oral glucose tolerance test, *prePregBMI* pre-pregnancy BMI, *TOP* Treatment Of Obese Pregnant study, *VIF* variance inflation factor

In a linear regression model adjusted for maternal age, educational level, smoking during pregnancy, prePregBMI, GWG, TOP intervention assignment, offspring sex, GA, and cell-type composition (Model 2b), maternal insulin levels were associated with cord blood DNAm at two sites: cg03310479 [β = -0,42(95%CI:-0.57;-0.25), *P* = 2.7E-07] and cg02746691 [β = 0.43(95%CI:0.31;0.62), *P* = 2.6E-07] annotated to *C11orf54* and *RARB,* respectively (FDR < 10%) (Fig. 1C*, *Tables S1 and S2). These two DNAm sites are located within putative transcription factor (TF) binding sites according to JASPAR database (accessed Sep.2024) [22]. The cg03310479 in *C11orf54* is in a putative binding site for EGR1, EGR2, EGR3, EGR4, KLF17, RREB1, PRDM9, and SP8 and the cg02746691 in *RARB* is in a putative binding site for E2F6, TFDP1, GLIS2, ZBTB24, ZNF682, and E2F8 (*P* < 10^–4^) (Table S3). We further used the GWAS Catalog and Gtex Portal (accessed Sep.2024) and EWAS Catalog and EWAS Atlas (accessed Dec.2024) to investigate the biological role of *C11orf54* and *RARB* and the respective CpG sites. We found that both genes are moderately expressed in visceral adipose tissue with 9.4 and 5.8 transcripts per million for *C11orf54* and *RARB*, respectively (Gtex Portal). While the GWAS Catalog did not report associations between *C11orf54* and metabolic phenotypes, several genetic variants in *RARB* have been linked to metabolic traits: rs1435703 was associated with obesity (GCST000426), rs76532059 with fasting insulin levels in childhood (GCST90310278), rs138514634 with abdominal fat cell number (GCST90104785), and rs322699 with abdominal fat distribution (GCST90020028). We also conducted a search in the EWAS Catalog and EWAS Atlas that showed that DNAm of both genes has been already associated with relevant metabolic traits such as insulin resistance for *C11orf54* and several metabolic traits like obesity, incident and prevalent T2D, lipids concentrations and non-alcoholic fatty liver disease for *RARB*, as extensively reported in Table S5.

However, neither of the two methylation sites (cg03310479 and cg02746691) were significantly associated with the abdominal/total FMr (r = − 0.192, *P* = 0.056 for cg03310479 and r = 0.122, *P* = 0.228 for cg02746691) in our cohort.

## Discussion

Fasting plasma maternal insulin levels at 18-20gw in pregnant mothers with obesity were associated with both the abdominal/total FMr in newborns and cord blood DNAm at two sites annotated to *RARB* and *C11orf54* genes after adjustment for multiple maternal metabolic risk factors and potential confounders. These findings support that maternal glucose metabolism during mid-pregnancy at 18–20gw plays a significant role in fetal fat distribution and epigenetic modifications, highlighting potential metabolic programming effects. It is noteworthy that *RARB* encodes retinoic acid receptor beta. It binds retinoic acid (RA), vitamin A in its biologically active form, which mediates cellular signaling in embryonic morphogenesis, cell growth and differentiation [23]. RA is important for energy metabolism and the interaction with other transcriptional factors may contribute to the short-term dynamic regulation of hepatic *Gck* expression in response to insulin and macronutrients intake [23, 24]. Several studies have demonstrated that RA conveys anti-obesity and anti-lipogenic properties via transcriptional regulation of multiple genes, mainly in liver and adipose tissue [23, 24]. Additionally, vitamin A plays an essential role in pancreatic endocrine development, where deficiency can decrease β-cell mass and impair glucose tolerance in adulthood [25]. Rarβ knockout in embryonic stem cells diminished the pancreatic endocrine differentiation and reduced expression of *Pdx1*, *Gcg*, *Iapp*, and *Ins1*, while overexpression of Rarβ increased insulin secretion, in the presence of all-trans-retinoic acid [25]. Consistently, epidemiological studies reported associations between vitamin A deficiency and the risk of T2D [26].

Although less is known about the protein coding gene *C11orf54*, its ortholog Meep enables hydrolase activity, acting on ester bonds and zinc ion binding activity, and has been involved in cellular response to glucose stimulus and positive regulation of insulin receptor signaling in Drosophila melanogaster [27], specifically required in the developing fat body to tolerate a high-sugar diet. Additionally, expression of the mouse ortholog correlated positively with obesity in insulin resistant mice [27, 28]. Although the potential involvement in insulin signaling pathways, further research is needed to elucidate the specific functions of *C11orf54* in humans.

Both cg03310479 (*C11orf54*) and cg02746691 (*RARB*) are located within putative TFs binding sites and changes in the methylation status at single CpG sites may affect their binding affinity [29]. Some of the binding TFs seem to affect insulin signaling and/or adipogenesis (Table S4). EGR1 and EGR2 play a key role in insulin signaling and lipid metabolism and have been involved in adipocyte differentiation programs [30], and RREB1 has been shown as novel candidate gene for diabetes affecting insulin sensitivity, beta-cell function and adipogenesis [31] (Table S4). The goal of this analysis was to uncover potential regulatory mechanisms of DNAm on gene function. However, functional studies are needed to directly assess how DNAm at these sites affects TFs binding and gene regulation.

Tobi et al. previously investigated in a meta-analysis associations between cord blood DNAm and maternal insulin [19]. However, no CpGs reached significance after multiple testing correction based on FDR < 10% and no robust DMRs were identified. Among our sites with FDR < 10%, the cg03310479 in *C11orf54* was not available on the EPIC array and the cg02746691 in *RARB* showed no association in their results based on *P* < 0.05. Nonetheless, we acknowledge substantial differences in methodology and population characteristics, including the unique RCT design of our study involving women with obesity, the comprehensive adjustment for confounders in our models, and the earlier timing of fasting insulin measurements.

Waldrop et al. have previously studied associations between DNAm, maternal insulin and offspring body composition, but insulin was measured at 27-34gw in pregnant mothers recruited from the general population and offspring’s body composition was assessed via air displacement plethysmography, giving no chance for abdominal body composition assessment [17]. In line with our study, where no significant associations were observed between maternal insulin-associated DNAm sites and offspring abdominal adiposity, they found 30 differential methylated regions (DMRs) associated with maternal insulin, but these DMRs were not associated with newborn, infant, nor child adiposity outcomes [17]. Taken together, these findings suggest that maternal insulin and newborn adiposity associations may not be linked through cord blood DNAm [17]. Other epigenetic mechanisms may account for these associations, or it is also possible that these links may underline direct effects of insulin and glucose availability on fetal adipose depots or indirect effects through other fuels (e.g., triglycerides) [13, 17]. Noteworthy, *C11orf54* DNAm was nominally associated with abdominal/total FMr (*P* = 0.056), with the direction of the association aligning with the findings from the maternal insulin and abdominal/total FMr association analysis. Thus, larger studies are needed to further explore associations between cord blood DNAm and regional adiposity in offspring and to confirm our findings.

In line with our association between maternal insulin at 18–20gw and abdominal/total FMr, insulin has been shown as an important determinant of placental metabolic homeostasis, both glycemic and lipidic, in the first trimester. Insulin and IGF-2 activate the insulin receptor, altering placental metabolism and stimulating triglyceride accumulation, also in euglycemic conditions, which may explain prevalence of excess adiposity [11]. Insulin can regulate placental glucose transport through GLUT4 trafficking [32], can affect the fetoplacental vasculature through activation of the MAPK pathways and secretion of endothelin leading to vasoconstriction, and can affect lipid metabolism leading to lipotoxicity and impair placental function due to increased oxidative stress, reduced angiogenesis and inflammation [33]. So, the early hyperinsulinemia that characterizes a higher percentage of woman with obesity creates a metabolic environment of excess nutrients and low-grade inflammation. This environment influences the development of the fetoplacental unit, potentially contributing to the fetal metabolic programming and impacting neonatal fat composition and future cardiometabolic risk [7, 33]. While maternal glycemia is a recognized key driver of neonatal adiposity, especially during later pregnancy [6, 10, 14], hyperinsulinemia also warrants attention. It may influence adverse offspring outcomes in pregnant women with obesity [7, 13, 14] and might not be fully addressed by current screening methods or by the management of maternal hyperglycemia alone in clinical practice [7]. Notably, hyperinsulinemia influences the production of other maternal fuels that play a crucial role in fetal growth and impact offspring fat deposition [10, 11, 13, 17].

The strengths of our study are the prospective RCT design allowing for longitudinal data collection over time, including detailed measures of regional adiposity in newborn using DXA and the higher pre-pregnancy BMI of our cohort making it likely that women will enter pregnancy with higher insulin values. Abdominal/total FMr reflects more accurately the abdominal fat distribution compared to the total abdominal adiposity and may be more important for cardiometabolic health later in life. Our study has some limitations: the use of the 450 k array, despite the availability of more advanced arrays with higher coverage, and the lack of differential methylated regions analysis. Although fasting insulin levels provide significant insight into the metabolic status of pregnant women, the lack of fasting blood glucose measures to calculate HOMA-IR or the lack of other dynamic measures of insulin resistance is a limitation of our study. Other limitations are the use of FDR threshold < 10%, which may increase the risk of false-positive findings, and the lack of replication in larger cohorts. Replication of our findings in independent published cohorts was not feasible due to the unavailability of comparable insulin measures and CpG coverage, as well as differences in study design and analytical methods. Further studies comparing offspring of women with normal weight and obesity are needed to assess differences in biological pathways, to verify strengths of association and metabolic risks. Additionally, functional studies of the identified methylation sites are essential to elucidate the biological mechanisms potentially involved.

## Conclusion

Among different maternal metabolic risk factors, fasting maternal insulin levels in pregnant mothers with obesity at 18-20gw were associated with the abdominal/total FMr in newborns, a known predictor of cardiometabolic risk later in life. Maternal insulin was also associated with cord blood DNAm at two sites annotated to genes that could be potentially involved in metabolic programming. Further studies are needed to dissect the possible mechanisms underlying the link between early maternal insulin status and offspring adiposity composition and the potential mediation through other maternal fuels availability.

## Supplementary Information

Supplementary Material 1

Supplementary Material 2

## Data Availability

DNA methylation data from cord blood of the TOP-study (accession number LUDC2020.08.14) are deposited in the Lund University Diabetes Centre repository (https://www.ludc.lu.se/resources/repository) and are available to academic researchers upon request through the repository portal. However, due to EU and national legislation, genome-wide raw data and individual level clinical data are not available.
