# Supplementary material for: Insulin levels at 18–20 gestational weeks in pregnant women with obesity are associated with newborn abdominal fat deposition and DNA methylation in cord blood
Source: Clin Epigenetics. 2025 Jul 11;17:123. doi: 10.1186/s13148-025-01923-y (PMC12255083; doi:10.1186/s13148-025-01923-y)
Supplement: Supplementary file 1 — Supplementary Material 1 [file 13148_2025_1923_MOESM1_ESM.docx]

**SUPPLEMENTARY FILE for**

**Insulin levels at 18-20 gestational weeks in pregnant women with obesity associate with newborn abdominal fat deposition and DNA methylation in cord blood.**

*Alice Maguolo^1^, Josefine Jönsson^1^, Alexander Perfilyev^1^, Allan Vaag^2,3^, Emma Malchau Carlsen ^4,5^, Kirsten Nørgaard ^3,6^, Paul W. Franks^2,7^, Kristina M. Renault^6,8^*, Charlotte Ling^1^*.*

***Corresponding authors****:*

*Alice Maguolo (alice.maguolo@med.lu.se) ORCID 0000-0002-8921-2012*

*Charlotte Ling (charlotte.ling@med.lu.se) ORCID 0000-0003-0587-7154*

**Methods**

1. Research cohort

From the Treatment of Obese Pregnant women (TOP)-study, 232 mother-child pairs with available DNAm in CB were included. The TOP-study is a randomized controlled trial of 425 pregnant women with obesity (BMI>30 kg/m^2^) including two lifestyle intervention groups. Pregnant mothers were randomized to one arm with physical activity assessed with pedometer, one arm with physical activity plus dietary advice, and a control arm receiving standard of care (1,2). After considering miscarriages, withdrawing from the study, or moving from the region, 389 women completed the study. Maternal age, maternal educational level, pre-pregnancy BMI (kg/m2), gestational weight gain (GWG) (kg), smoking during pregnancy, offspring sex, gestational age (weeks), and body composition measurements were considered for this study.

Maternal age, pre-pregnancy BMI, and maternal educational level were recorded at enrollment (gestational weeks 11–14). Smoking during pregnancy was acquired through medical records, pre-pregnancy BMI by self-reported weight, and GWG was determined by subtracting pre-pregnancy weight with weight measured at 36-37 weeks (1). At 18-20 gestational weeks, fasting maternal insulin (pmol/L), c-peptide (pmol/L), and 2-h plasma glucose (mmol/L) during 75-g, 2-hour standard oral glucose tolerance test (OGTT) has been measured, as previously described (1). Unfortunately, no fasting glucose measurements are available for this cohort. Serum levels of insulin and c-peptide were analyzed at the Clinical Biochemistry Department, Hvidovre University Hospital, using Immulite® 1000 Analyzer (Diagnostic Products Corporation, Los Angeles, CA, USA) with kits from Siemens Healthcare, Germany, after excluding hemolyzed samples.

At birth anthropometric data, including measures of body composition assessed by dual-energy X-ray absorptiometry (DXA) scan (DXA, Hologic 4500, Bedford, MA, USA) in newborns within 48h from birth, were collected (3). The estimation of abdominal fat mass and fat-free mass has been previously described (3). The abdominal/fat mass ratio (FMr) was calculated as abdominal fat mass (g)/total fat mass (g) assessed by DXA.

Detailed information on enrollment, conduction of the trial, and clinical measurements are extensively described elsewhere (1,3–5).

2. DNA methylation analysis and bioinformatics pipeline

Cord blood was collected from the umbilical vein of the clamped umbilical cord at birth. Samples were frozen (-80°C) and stored at the Copenhagen University Hospital Hvidovre biobank. The Gentra Puregene Blood Kit (Qiagen) was used for DNA extraction, according to the manufacturer's instructions. DNA concentration and purity were determined using NanoDrop (NanoDrop Technologies, Inc.). Bisulfite conversion of genomic DNA was performed using the EZ-96 DNA methylation kit (Zymo Research Corporation, Irvine, CA).

Genome-wide DNA methylation analysis was performed using Illumina Infinium HumanMethylation450 BeadChips (Illumina, San Diego, CA), covering 485,577 sites, as previously described(6).

Methylation data were acquired from 460,729 probes. We filtered out 1,594 probes with mean detection P-value≥0.01, 65 rs-probes, 3,091 ch-probes targeting non-CpG sites, 416 Y-chromosome probes, 14,466 cross-reactive probes, and 5,216 polymorphic probes with a minor allele frequency >0.1 (7). Background correction and Beta-Mixture Quantile normalization were performed (8), and ComBat corrected for batch effects (9). Since cord blood contains multiple cells, a reference-based method was employed to correct for any potential effects of cellular heterogeneity(10). This deconvolution technique allows to estimate relative proportions of CB cell types using CB-derived signatures of CD8T, CD4T, natural killer, B-cells, monocytes, and neutrophils DNA methylation.

3. Statistical analysis

The statistical analyses were performed on 158 mother-child pairs out of 232, as 21 samples were removed for failed entry QC, bisulfite conversion, sex mismatch, and 53 for missing data regarding maternal insulin or other covariates used in the regression models, as showed in detail in *Figure 1A*. The characteristics of the 158 mother-child pairs used for analysis are described in *Figure 1B*.

For genome-wide bioinformatic and statistical analyses, β-values were converted into M-values, M=log2(β/[1-β]), to eliminate heteroscedasticity(11). Educational Level was categorized into three categories: 1) Grammar school 10 years; 2) Secondary school 12 years, Vocational training school, and Further education 1-2 years; 3) Tertiary education 3-4 years (Bachelor level) and Advanced education (post-graduate).

Statistical analyses were performed using the R Project for Statistical Computing Software v4.2.1 (2022-06-23) and the IBM SPSS Statistics v29. Data are presented as mean ± SD, unless stated otherwise. The 95% confidence intervals (CIs) for the standardized beta coefficients were calculated by scaling the 95% CIs of the unstandardized beta coefficients.

3.1a Associations between abdominal/total FMr and maternal insulin

Spearman’s correlation was used to assess bivariate associations between abdominal/total FMr and both fasting maternal c-peptide and insulin. C-peptide and insulin, both independent markers of beta cell’s function, were used to exclude potential variability in insulin dynamics. Multivariable linear regression was performed to assess associations between maternal insulin and newborn abdominal/total FMr, independent of maternal age, educational level, smoking during pregnancy, pre-pregnancy BMI, GWG, TOP intervention assignment, GA, and offspring sex (Model 1a, Figure 1a) + 2h-OGTT glucose (Model 1b). Sex was previously found to affect insulin’s effect on adiposity (12,13). To investigate whether the association between maternal insulin levels and newborn abdominal/total fat mass ratio (FMr) differs across offspring sex, an interaction analysis was conducted by including a multiplicative interaction term insulin*sex in the multivariable linear regression model (Model 1c). This approach allowed for an examination of the potential effect of sex on insulin effects without reducing the sample size or compromising the robustness of the model.

3.1b Associations between maternal insulin and cord blood DNAm

Multivariable linear regression was performed to assess associations between maternal insulin and cord blood DNAm, adjusted for the same covariates as above (Model 2a) + cell-type composition (Model 2b), as shown in *Figure 1A*. Associations between maternal insulin and cord blood DNAm were corrected for multiple testing using false discovery rate (FDR) analysis (Benjamini-Hochberg), and associations with FDR<10% are presented (Figure 1C).

3.1c Associations between maternal insulin and cord blood DNAm

Pearson’s correlation was used to assess bivariate associations between abdominal/total FMr and the M values of the two methylation sites cg03310479 and cg02746691.

REFERENCES

1. Renault KM, Nørgaard K, Nilas L, Carlsen EM, Cortes D, Pryds O, et al. The Treatment of Obese Pregnant Women (TOP) study: a randomized controlled trial of the effect of physical activity intervention assessed by pedometer with or without dietary intervention in obese pregnant women. Am J Obstet Gynecol. 2014 Feb;210(2):134.e1-9.

2. Renault K, Nørgaard K, Andreasen KR, Secher NJ, Nilas L. Physical activity during pregnancy in obese and normal-weight women as assessed by pedometer. Acta Obstet Gynecol Scand. 2010 Jul;89(7):956–61.

3. Carlsen EM, Renault KM, Nørgaard K, Nilas L, Jensen JEB, Hyldstrup L, et al. Newborn regional body composition is influenced by maternal obesity, gestational weight gain and the birthweight standard score. Acta Paediatr Oslo Nor 1992. 2014 Sep;103(9):939–45.

4. Ejlerskov KT, Christensen LB, Ritz C, Jensen SM, Mølgaard C, Michaelsen KF. The impact of early growth patterns and infant feeding on body composition at 3 years of age. Br J Nutr. 2015 Jul;114(2):316–27.

5. Berglund NR, Lewis JI, Michaelsen KF, Mølgaard C, Renault KM, Carlsen EM. Birthweight z-score and fat-free mass at birth predict body composition at 3 years in Danish children born from obese mothers. Acta Paediatr. 2022;111(7):1427–34.

6. Jönsson J, Renault KM, García-Calzón S, Perfilyev A, Estampador AC, Nørgaard K, et al. Lifestyle Intervention in Pregnant Women With Obesity Impacts Cord Blood DNA Methylation, Which Associates With Body Composition in the Offspring. Diabetes. 2021 Apr;70(4):854–66.

7. McCartney DL, Walker RM, Morris SW, McIntosh AM, Porteous DJ, Evans KL. Identification of polymorphic and off-target probe binding sites on the Illumina Infinium MethylationEPIC BeadChip. Genomics Data. 2016 Sep;9:22–4.

8. Teschendorff AE, Marabita F, Lechner M, Bartlett T, Tegner J, Gomez-Cabrero D, et al. A beta-mixture quantile normalization method for correcting probe design bias in Illumina Infinium 450 k DNA methylation data. Bioinforma Oxf Engl. 2013 Jan 15;29(2):189–96.

9. Johnson WE, Li C, Rabinovic A. Adjusting batch effects in microarray expression data using empirical Bayes methods. Biostat Oxf Engl. 2007 Jan;8(1):118–27.

10. Gervin K, Salas LA, Bakulski KM, van Zelm MC, Koestler DC, Wiencke JK, et al. Systematic evaluation and validation of reference and library selection methods for deconvolution of cord blood DNA methylation data. Clin Epigenetics. 2019 Aug 27;11(1):125.

11. Du P, Zhang X, Huang CC, Jafari N, Kibbe WA, Hou L, et al. Comparison of Beta-value and M-value methods for quantifying methylation levels by microarray analysis. BMC Bioinformatics. 2010 Nov 30;11(1):587.

12. Lima RA, Desoye G, Simmons D, Devlieger R, Galjaard S, Corcoy R, et al. The importance of maternal insulin resistance throughout pregnancy on neonatal adiposity. Paediatr Perinat Epidemiol. 2021 Jan;35(1):83–91.

13. Lima RA, Desoye G, Simmons D, Devlieger R, Galjaard S, Corcoy R, et al. Temporal relationships between maternal metabolic parameters with neonatal adiposity in women with obesity differ by neonatal sex: Secondary analysis of the DALI study. Pediatr Obes. 2020 Jul;15(7):e12628.
